# EVA-YOLOv8: an improved YOLOv8 model integrating multi-scale attention mechanism and vision transformer for multi-class road crack detection

**DOI:** 10.1038/s41598-026-46475-0

**Published:** 2026-04-06

**Authors:** Yao Liu, Fei Wang, Renjie Song, Yimin Wu, Hongbo Shen, Haiping Wu, Chao Zhang

**Affiliations:** 1 School of Civil Engineering, Central South University, Changsha, 410075 China; 2 National Engineering Laboratory for Construction Technology of High-Speed Railway, Central South University, Changsha, 410075 China; 3 Anhui Transport Consulting & Design Institute Co., Ltd., Hefei, 230088 China

**Keywords:** YOLOv8, Road crack detection, Attention mechanism, Vision Transformer, Lightweight network, Engineering, Mathematics and computing

## Abstract

Road crack detection is crucial for road maintenance and traffic safety. To address the low efficiency and limited generalization capability of traditional crack detection methods, this research proposes an improved model dubbed EVA-YOLOv8 (Efficient ViT Attention-YOLOv8) based on the YOLOv8n framework. This model integrates MobileViT Block and an Efficient Multi-scale Attention (EMA) mechanism, while employing the more efficient GhostConv to provide lightweight optimization for the network. Comparisons and ablation experiments were conducted on road crack datasets, and the attention mechanism of the model on crack features was analyzed using Grad CAM + + heatmap. Results indicate: (1) The EVA-YOLOv8 model achieved values of 0.897 (mAP@0.5), 0.706 (mAP@0.5:0.95), 0.907 (Precision), 0.881 (Recall), and 0.894 (F1-score), exhibiting good generalization ability across multiple categories, different scales, and low-contrast scenarios. (2) Compared with the original YOLOv8, the improved model demonstrates enhanced performance: mAP@0.5 and Recall are increased by 6.6% and 5.3%, respectively, while the parameters are reduced by 16.3%. (3) Grad-CAM + + heatmaps indicate that the model not only achieves high response values in the main region of the target but also performs well in target boundary localization and background suppression. This research may provide a reference for related fields.

**Supplementary Information:**

The online version contains supplementary material available at 10.1038/s41598-026-46475-0.

## Introduction

With the rapid development of China’s economy, urban transportation infra-structure construction has been continuously advancing, and roads play a critical role in urban traffic and regional connectivity^1^. However, during long-term service, road surfaces are prone to various types of distress—such as cracks, potholes, and spalling—caused by vehicular loads, environmental factors, and material aging^2^. Among these, cracks are the most common form of damage. If not detected and repaired in time, they can accelerate pavement deterioration, pose traffic safety risks, and increase maintenance costs^3^. Therefore, achieving efficient, accurate, and automated road crack detection is of great engineering significance for road maintenance and traffic safety^4,5^.

Traditional road crack detection methods mainly rely on manual inspection or semi-automatic image processing techniques. However, these approaches suffer from low efficiency, strong subjectivity, and vulnerability to illumination and surface texture interference^6–8^, making them inadequate for large-scale, rapid, and high-precision crack detection.

In recent years, with the rapid development of deep learning and computer vision, convolutional neural network (CNN)-based object detection methods have been widely applied in infrastructure defect detection. Liu et al.^9^ improved the YOLOv7 model by integrating the MobileViT lightweight network and the CBAM attention mechanism, achieving a mean average precision (mAP) of 87.9% in the detection of six typical PCB defects while reducing the computational cost. Cheng et al.^10^ proposed the SPSM-YOLOv8 model based on YOLOv8n, and optimized the model performance by replacing standard convolution with SPDConv, introducing the PSA mechanism, and adopting the SCDown downsampling mechanism. The model achieved a detection accuracy of 87.3% and a frame rate of 189 FPS on the RUOD underwater object recognition dataset, with its parameters and computation load reduced by 4.3% and 4.9% respectively compared with the baseline model.

In the field of road defect detection, Zhao et al.^11^ proposed the HWCNet semantic segmentation model fusing CNN and Transformer, which takes the Transformer-based AIFI module as the bottleneck and combines dense upsampling convolution with the ASFF structure in the decoder. The model achieved F1 scores of 88.65%, 76.73%, 88.78% and 86.04% on four asphalt pavement crack detection datasets respectively, showing good segmentation integrity and generalization ability. Liu et al.^12^ proposed the RIEC-YOLO model, which is based on YOLOv8 and adopts the RepViT-M1.5 lightweight backbone network, with the designed CN2C2f module and iEMA attention module. The model achieved an mAP50 of 87.0% on the RDD 2022 dataset, and its computational cost was only 16.9% of that of YOLOv8x. Gou et al.^13^ proposed the RoadNet road defect detection framework, which strengthened global feature modeling through a Transformer module embedded with multi-head self-attention (MHSA), and realized multi-scale feature fusion and joint capture of spatial-channel information by combining a multi-level feature pyramid network with the SCIM module. The framework achieved an mAP@0.5 of 0.9128 on the UAV-RDD dataset, with an inference speed of 210.01 ms per image on the CPU, making it suitable for the real-time detection of large-scale road defects by unmanned aerial vehicles.

YOLO series algorithms^14,16^ have exhibited remarkable performance in tasks such as crack recognition, pothole detection and concrete damage identification due to their advantages of end-to-end structure and real-time detection. However, in scenarios with limited computing resources, they still suffer from problems such as insufficient recognition rate, false detection and missed detection when dealing with fine crack features and complex pavement texture backgrounds^17,18^. To address this problem, this study carried out improvement research based on YOLOv8: the MobileViT module was embedded in the Backbone to strengthen the modeling capability of local and global features and improve the detection performance of fine and discontinuous cracks; the EMA mechanism was introduced in the Neck to dynamically adjust attention weights during the multi-scale feature fusion process, highlight the discriminative features of crack regions and suppress the interference of complex pavement backgrounds. Experimental results show that the MobileViT module and the EMA mechanism have a certain synergistic effect. The improved model enhances the recognition ability of fine-grained cracks in complex backgrounds while maintaining real-time detection performance, and thus has certain engineering application value.

## EVA-YOLOv8 road crack detection model

This section presents the overall architecture and key improvements of the pro-posed road crack detection model, EVA-YOLOv8 (Efficient ViT Attention-YOLOv8). Built upon the YOLOv8n framework, the model retains its efficient end-to-end detection pipeline, which consists of a back-bone network constructed with C2f modules, a neck structure based on FPN-PAN for multi-scale feature fusion, and a decoupled detection head.

### YOLOv8 algorithm

YOLOv8 (You Only Look Once, version 8) is a real-time object detection model proposed by the Ultralytics team in 2023^19,20^. It’ inherits the real-time performance of previous YOLO versions while achieving a better trade-off between accuracy and efficiency. YOLOv8 eliminates the traditional anchor-box mechanism and adopts an anchor-free design that directly predicts object centers. The decoupled classification and regression heads improve localization precision.

Its backbone network is based on an enhanced CSPDarknet53 architecture and incorporates C2f modules to strengthen multi-scale feature fusion. During training, YOLOv8 employs a Task-Aligned Assigner to dynamically match positive samples, and combines Distribution Focal Loss (DFL) for classification with CIoU + DFIoU dual regression losses, improving both convergence stability and bounding box regression accuracy.

**Fig. 1:**
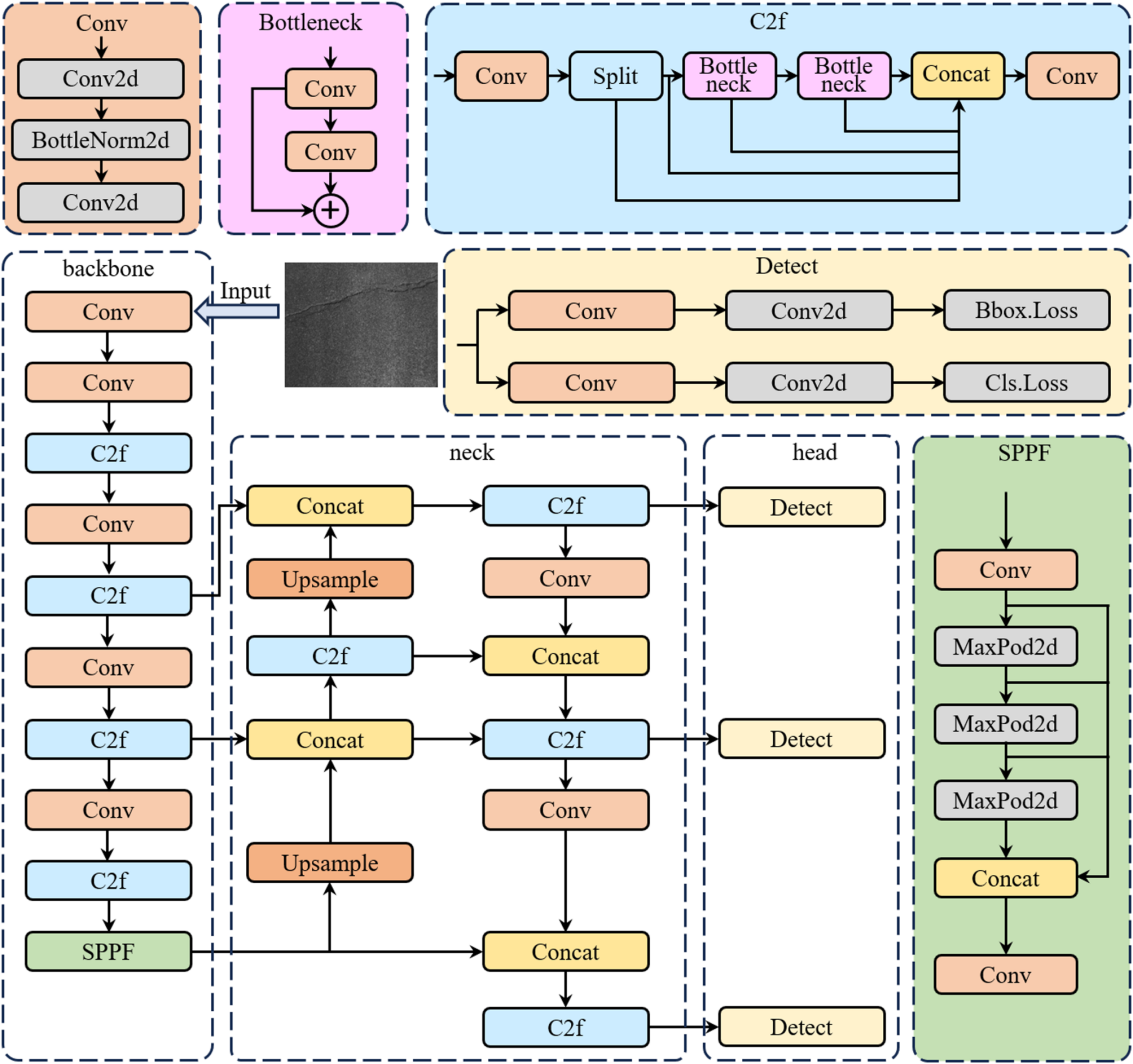
Overall architecture of the YOLOv8n algorithm.

Compared with earlier versions such as YOLOv5, YOLOv8 achieves significant improvements in both detection accuracy and inference speed. The lightweight variants demonstrate excellent real-time performance in edge computing environments^21^, making the framework highly suitable for resource-constrained applications such as tunnel or pavement defect detection (Fig. 1).

### MobileViT block-enhanced backbone network

The MobileViT Block integrated into EVA-YOLOv8 represents a lightweight visual representation module that fuses Convolutional Neural Networks (CNN) with Vision Transformers (ViT)^22^, originally proposed by Apple. It aims to balance the CNN’s local feature extraction capability with the Transformer’s global modeling ability, making it particularly suitable for edge computing and mobile vision tasks.

In this research, the MobileViT variant is embedded within the convolutional backbone to overcome the limited global receptive field of conventional CNNs. The module first extracts local features using standard convolutions, then performs an unfold operation to convert feature maps into sequences, which are fed into a Transformer encoder to model long-range dependencies. The features are subsequently reconstructed via a fold operation. Compared with standard ViT models, the MobileViT module integrates the spatial inductive capability of CNN with the global receptive field capability of ViT while ensuring model lightweighting, thus enabling the model to operate under the condition of limited computing resources.

**Fig. 2:**
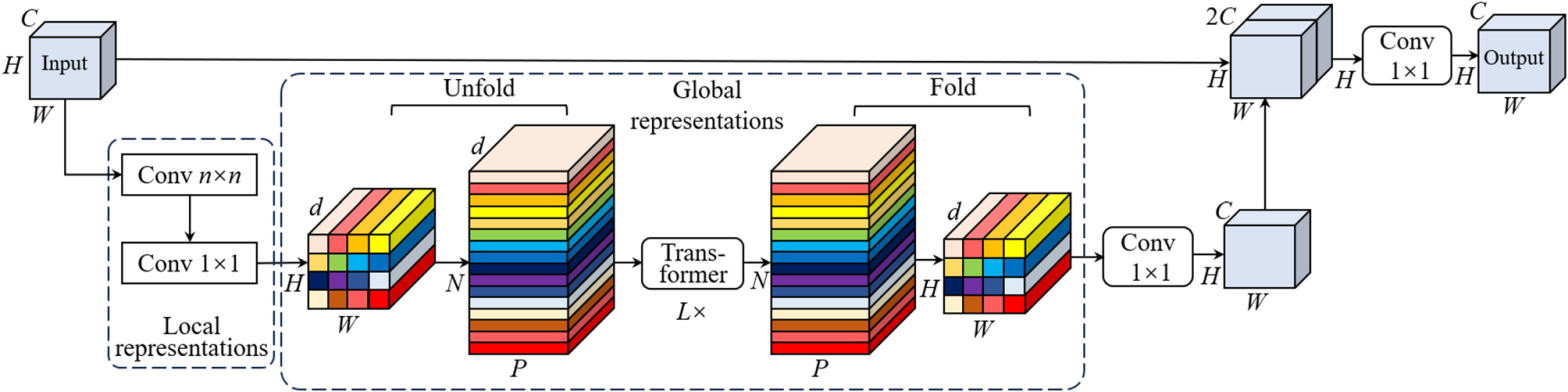
Structure of the MobileViT Block.

This structure effectively integrates local texture cues with global contextual semantics, which is especially advantageous for small-target detection in complex back-grounds—such as identifying fine and narrow cracks on road surfaces (Fig. 2).

### Efficient Multi-Scale Attention (EMA) Mechanism

**Fig. 3:**
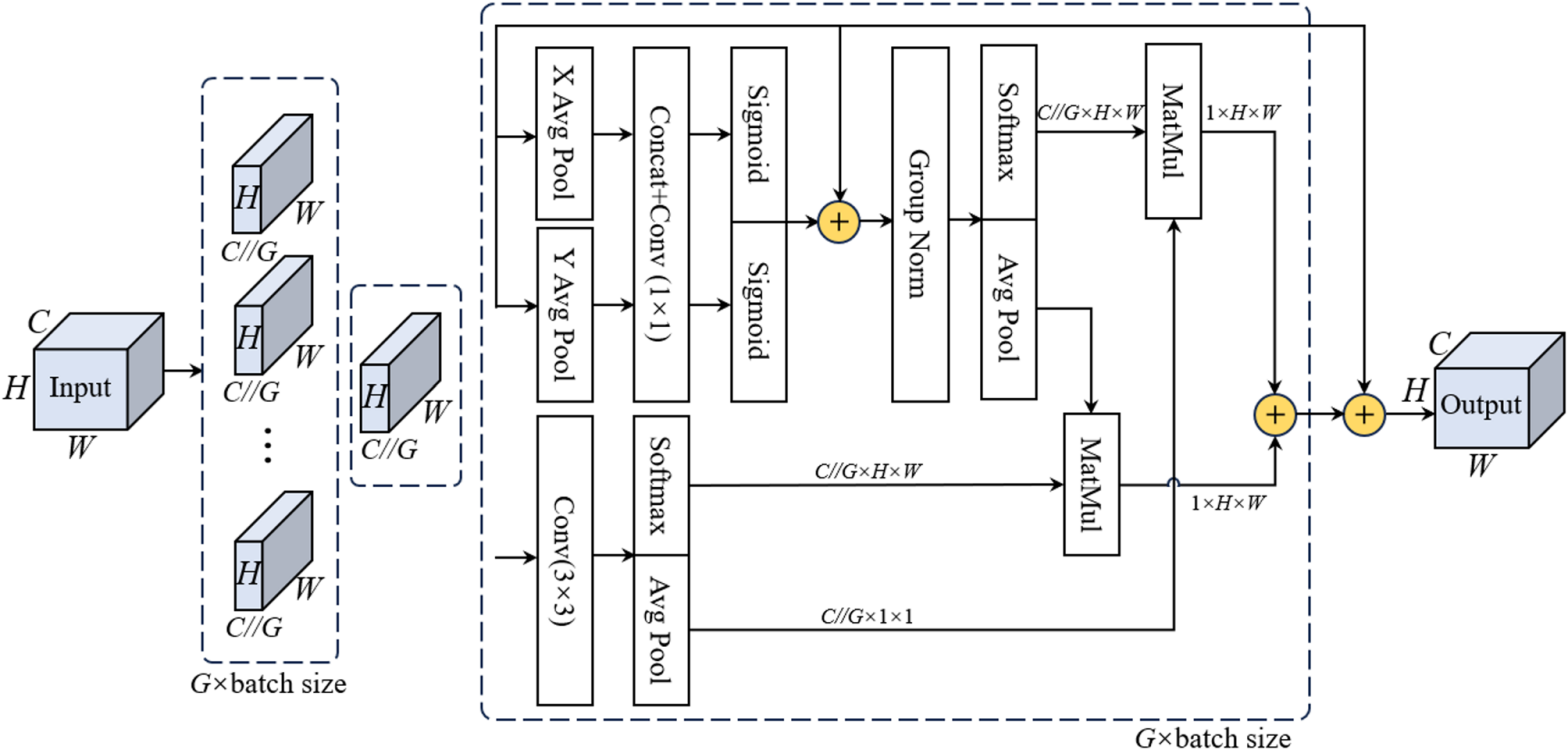
Structure of the EMA attention mechanism.

In road crack detection, target scales vary widely. To ensure the model performs consistently across different object sizes, the EMA mechanism is embedded in the neck network to enhance detection robustness and accuracy (Fig. 3).

EMA (Efficient Multi-scale Attention) is a high-performance attention mechanism^23^ that captures both local and long-range dependencies using convolutional kernels of varying receptive fields, while integrating global spatial information through non-linear adaptive weighting. This allows the module to preserve precise spatial localization while enhancing global contextual representation. Formally, the two-dimensional global average pooling operation can be expressed as:

1 $${z_c}=\frac{1}{{H \times W}}\sum\limits_{j}^{H} {\sum\limits_{i}^{W} {{x_c}(i,j)} }$$

where ${z_c}$denotes the average value of the *c*-th channel after global pooling, *H* and *W* represent the spatial dimensions, and ${x_c}$ is the feature value at spatial location (*i*, *j*) in channel *c*.

In the EMA module, this global spatial encoding from the 1 × 1 branch is combined with multi-scale convolutional responses to generate adaptive attention weights, enabling the network to focus on discriminative crack regions.

### GhostConv Module

The introduction of the MobileViT Block and EMA modules inevitably increases the model’s parameter count and computational cost. To balance model complexity and efficiency, the GhostConv module is employed to replace certain convolutional layers in the backbone network^24^.

The design of GhostConv stems from the observation that many feature maps in CNNs are highly redundant. Its core idea is to use inexpensive linear transformations to generate additional “ghost” feature maps from a small number of intrinsic features, thereby reducing redundant computation and memory usage without sacrificing representational power.

Specifically, GhostConv decomposes standard convolution into two stages: first, a regular convolution with reduced output channels generates a subset of intrinsic features; then, efficient linear operations—such as depth wise convolutions—are applied to produce additional ghost features that mimic the original ones. Experimental results indicate that incorporating GhostConv can substantially accelerate inference and re-duce parameter count and FLOPs, while maintaining nearly identical detection accuracy.

**Fig. 4:**
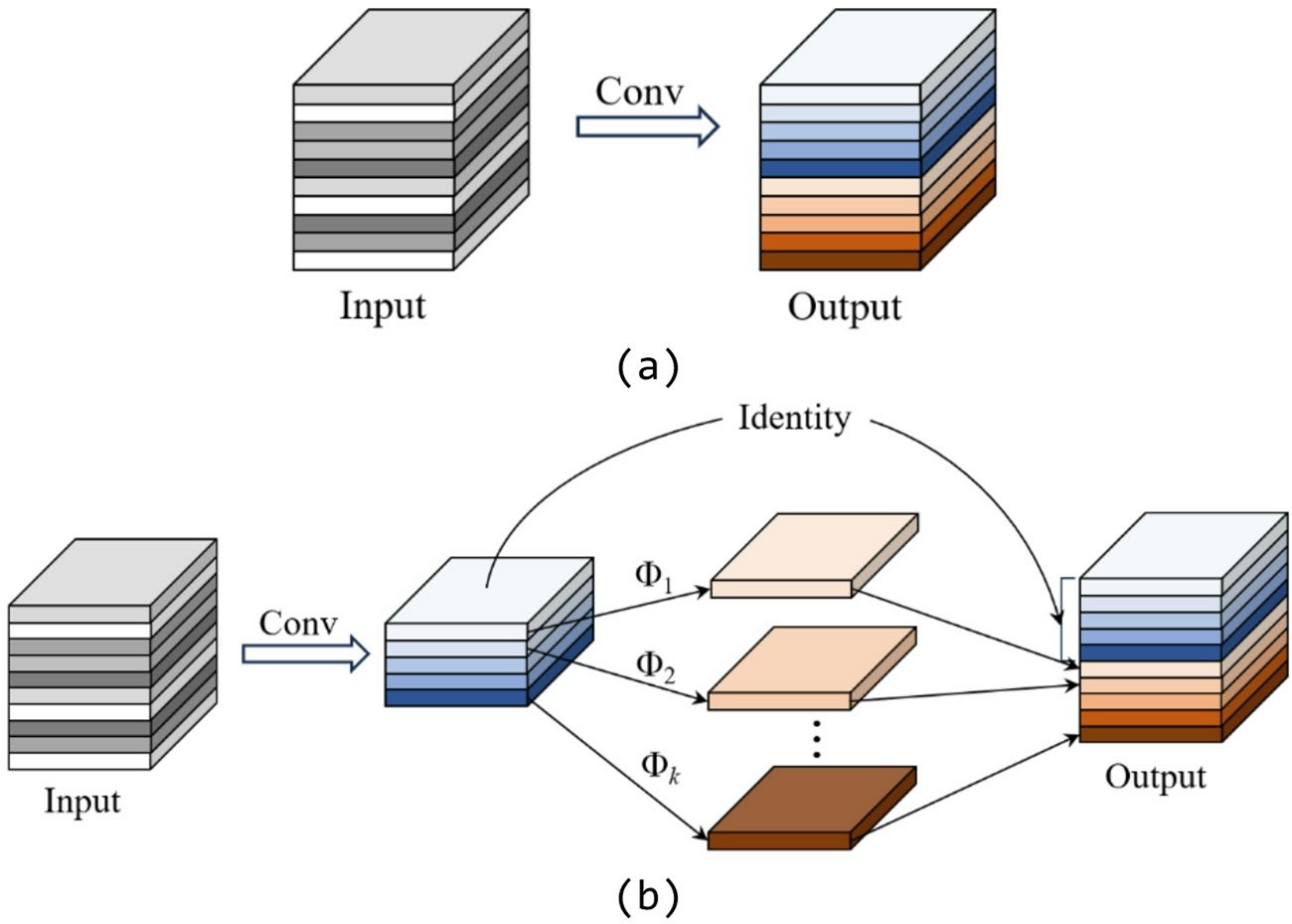
Standard convolution and Ghost convolution. (**a**) Standard convolution, (**b**) Ghost convolution.

This optimization enables EVA-YOLOv8 to retain high detection performance while remaining lightweight and suitable for real-time deployment on edge devices (Fig. 4).

**Fig. 5:**
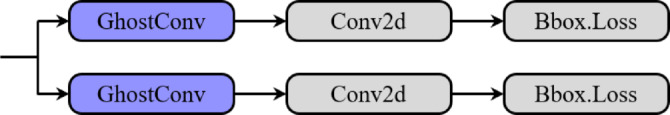
Comparison between standard convolution and Ghost convolution.

To improve the inference efficiency of the detection head, the original convolutional layers in the Head module are replaced with GhostConv, thereby reducing its parameter count and computational cost (Fig. 5).

### Overall architecture of the EVA-YOLOv8 model

To address the challenges of crack detection—such as small target size, irregular morphology, and cross-scale distribution—the proposed model introduces three major structural enhancements. First, a MobileViT Block is embedded into the backbone to strengthen both local and global feature modeling. Second, an Efficient Multi-scale Attention (EMA) mechanism is incorporated into the neck to adaptively adjust attention weights during multi-scale feature fusion. This design suppresses background noise and emphasizes salient crack regions, thereby enhancing feature discriminability and robustness. Finally, a Ghost-Conv lightweight convolution module replaces part of the standard convolution layers, reducing redundant feature computations and overall model complexity. This modification significantly decreases parameter count and computational cost while maintaining detection accuracy, improving the model’s real-time performance and deployability.

**Fig. 6:**
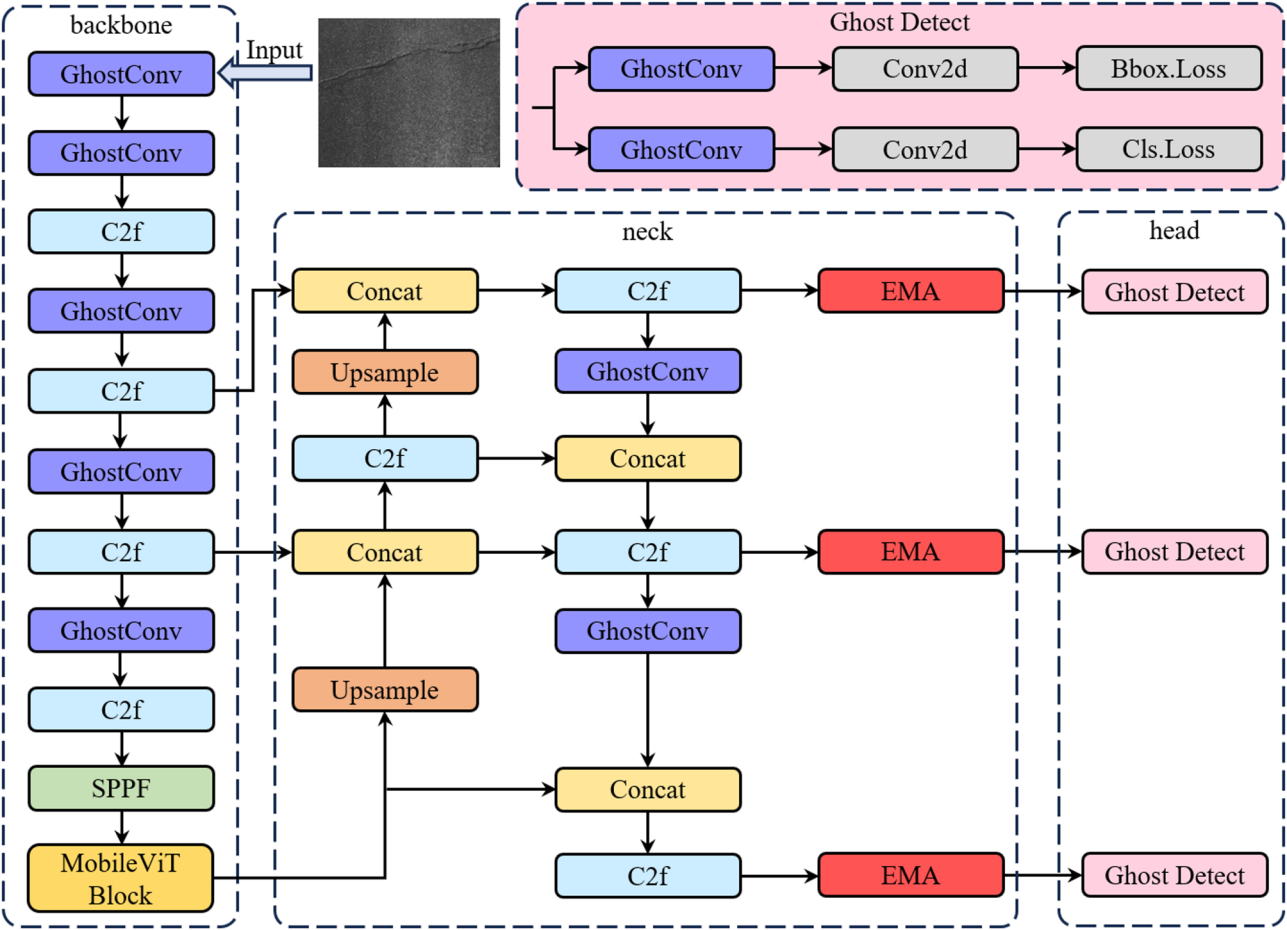
Overall architecture of the EVA-YOLOv8 algorithm.

In summary, EVA-YOLOv8 achieves an effective balance between global perception, multi-scale feature fusion, and lightweight design. It simultaneously ensures high detection accuracy and computational efficiency, providing a more robust and deployable solution for complex road crack detection scenarios (Fig. 6).

## Construction of road crack dataset

### Road crack image acquisition

**Fig. 7:**
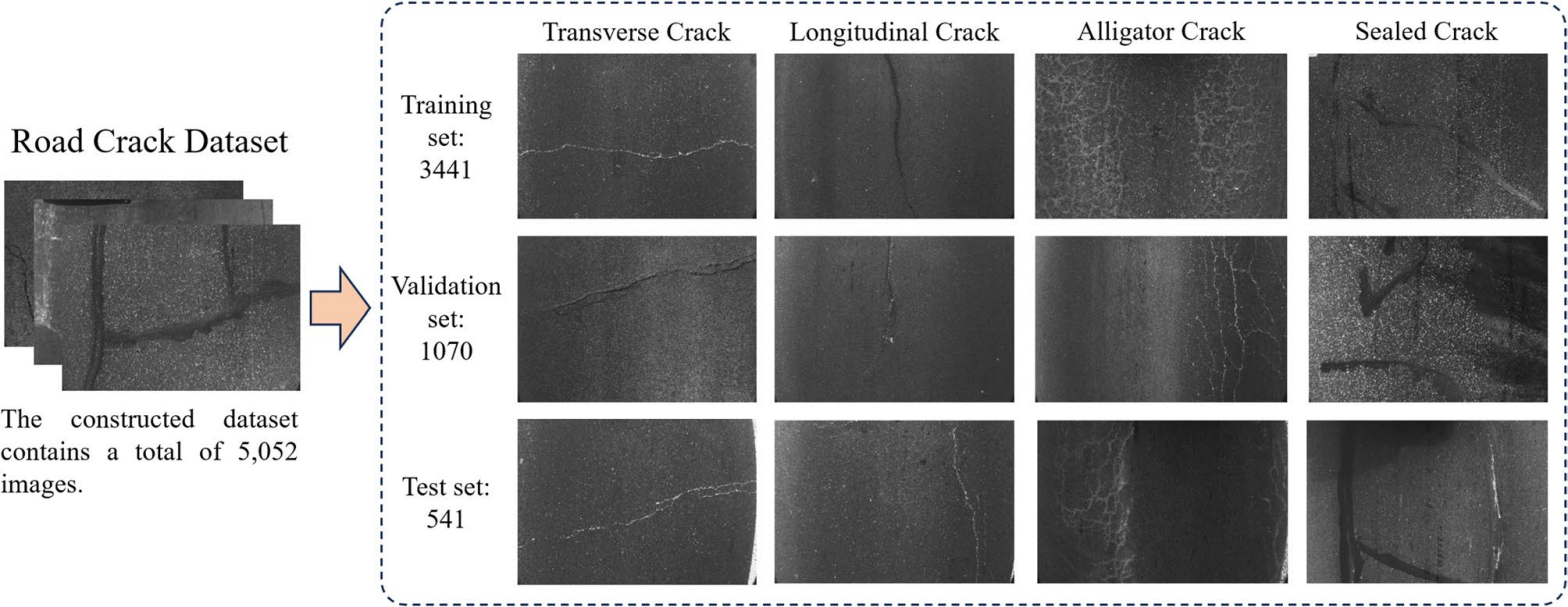
Examples of road crack dataset.

The road crack dataset used in this research mainly comes from the Central South University Transportation Intelligent Low Carbon Construction and Maintenance and Intelligent Safety Control System Pilot Base and publicly available road crack datasets^25,26^. This multi-source data collection strategy enables the acquisition of diverse and comprehensive image samples, which is beneficial for improving the generalization capability of the trained model. The dataset consists of 5052 images, including 3441 for training, 1070 for validation, and 541 for testing. Representative road crack images are shown in Fig. 7. The collected dataset includes four typical categories of cracks: Transverse Crack, Longitudinal Crack, Alligator Crack, and Sealed Crack, which comprehensively cover the common defect types observed on pavement surfaces.

### Data augmentation

**Fig. 8:**
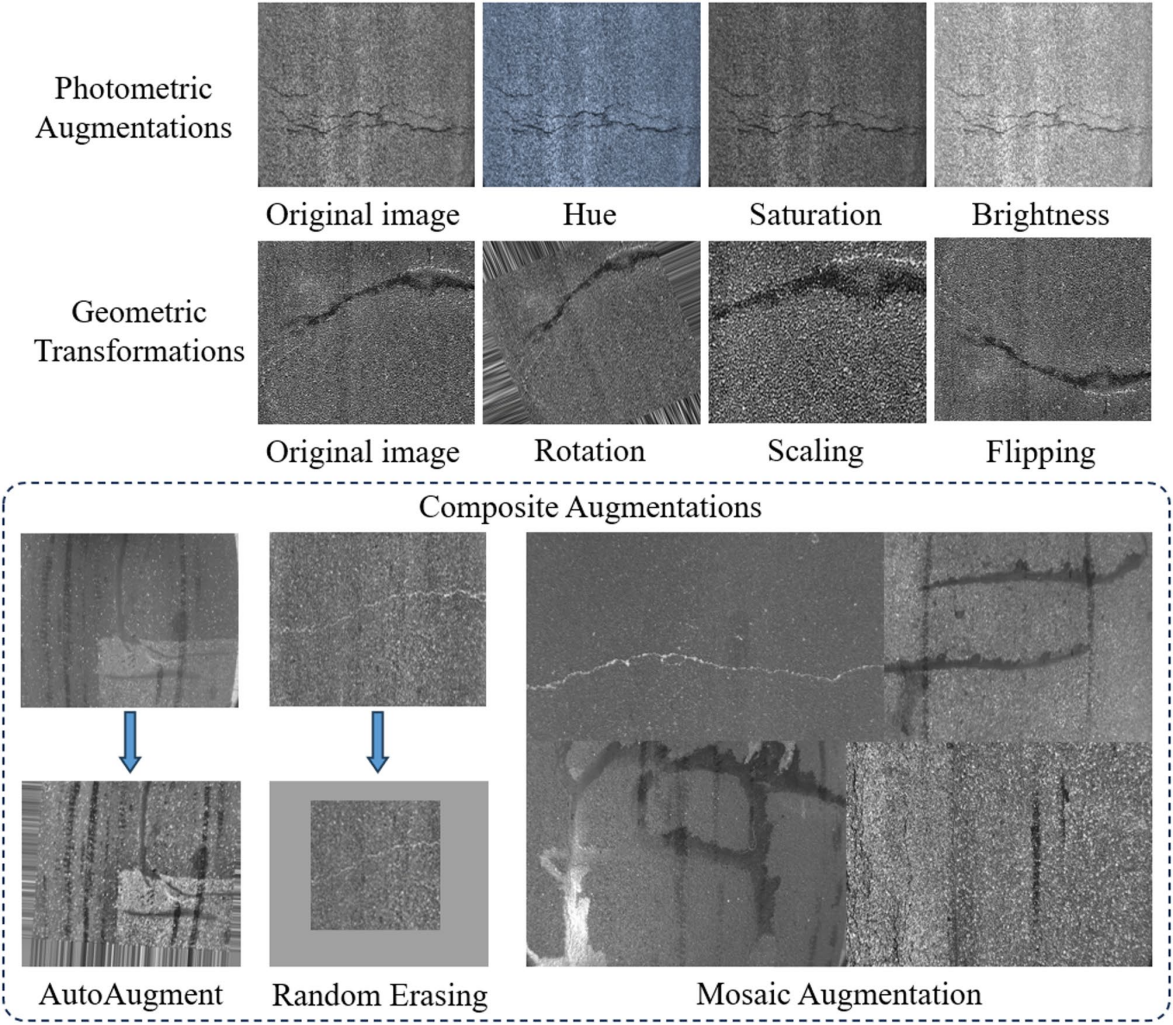
Visualization of dataset augmentation strategies.

To enhance the model’s generalization capability across diverse scenarios, a series of data augmentation strategies were applied during training. Specifically, color perturbations were introduced, including hue adjustment (± 10%), saturation variation (± 70%), and brightness adjustment (up to 40%). Geometric transformations such as translation (10%), scaling (50%), and random horizontal flipping (probability of 50%) were also employed, along with mosaic augmentation to enrich spatial diversity. In addition, automatic augmentation strategies and random erasing (probability of 40%) were incorporated to further increase the variability of training samples. The integration of these diverse augmentation techniques effectively improves the model’s robustness and adaptability in complex and heterogeneous environments. An overview of the applied augmentation strategies is shown in Fig. 8, illustrating the diversity introduced to the dataset.

### Data annotation

**Fig. 9:**
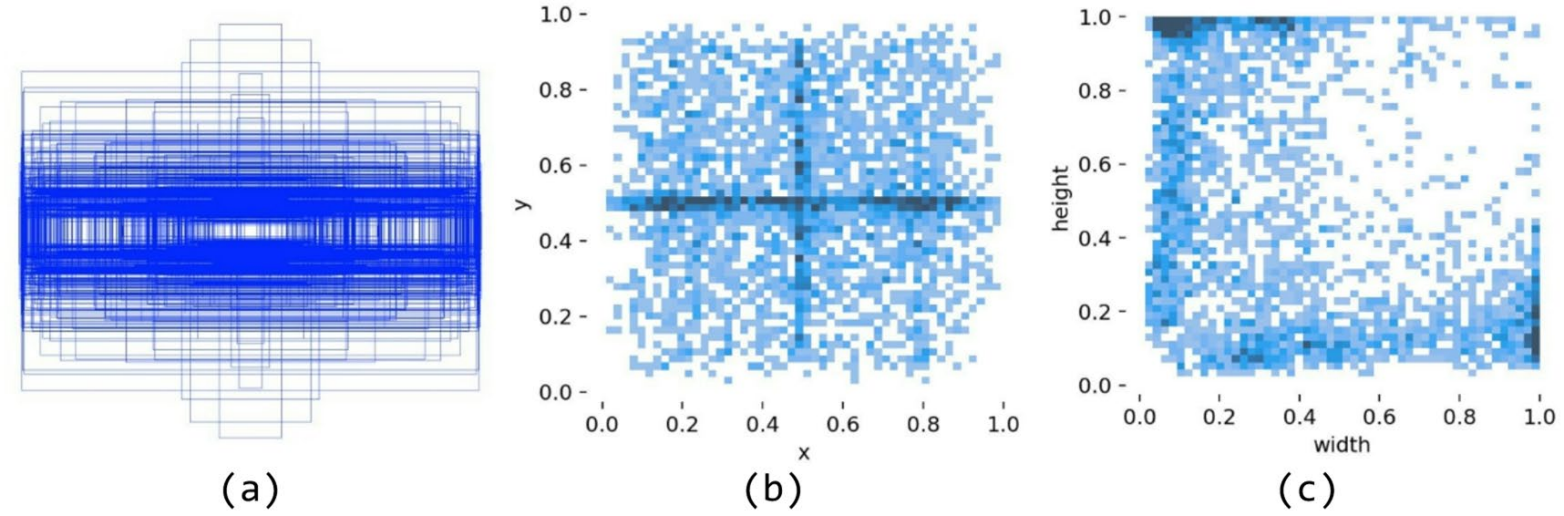
Annotation statistics of the dataset. (**a**) Bounding box distribution, (**b**) Center point distribution, (**c**) Target proportion.

For data labeling, bounding boxes were manually drawn around cracks such as longitudinal, transverse, and alligator types, with the corresponding class labels recorded. Considering the slender and discontinuous characteristics of cracks, the bounding boxes were required to tightly enclose the target. Cracks longer than 50 pixels were fully covered, while for those narrower than 3 pixels, a slight relaxation was allowed to ensure complete inclusion. The annotations were saved in the YOLO format (.txt) containing the class ID and normalized coordinates, ensuring compatibility with the training framework. The dataset annotation examples are illustrated in Fig. 9.

This section may be divided by subheadings. It should provide a concise and precise description of the experimental results, their interpretation, as well as the experimental conclusions that can be drawn.

## Model training

### Experimental environment

All experiments were conducted on a system running Windows 10 Professional, equipped with a 12th Gen Intel(R) Core(TM) i9-12900KS CPU (3.4 GHz), 64 GB RAM, and an NVIDIA GeForce RTX 4070 SUPER GPU. The model was implemented using the PyTorch 2.3.1 framework with CUDA 12.6 acceleration. The detailed experimental environment configuration is presented in Table 1.

**Table 1:** Experimental environment parameters.

Parameter	Setting
epoch	300
batch	32
imgsz	960
workers	8
lr0	0.01
momentum	0.937
weight_decay	0.0005

### Loss function

The default bounding box regression loss in YOLOv8 is the Complete Intersection over Union (CIoU) loss^27,28^, which comprehensively measures the geometric relationship between the predicted and ground-truth boxes by incorporating the IoU, center distance, and aspect-ratio constraints. Although CIoU improves localization accuracy, its performance heavily depends on the quality of positive–negative sample matching. The heuristic assignment strategy in YOLOv8 may fail to achieve global optimal matching, and when no overlap exists between the predicted and ground-truth boxes, the loss can suffer from gradient vanishing. Moreover, in dense small-target detection scenarios such as crack identification, the aspect-ratio constraint of CIoU has limited effect, potentially leading to unstable localization.

To address these shortcomings, the loss function is improved in this study. The Hungarian Matching algorithm^29,30^ is introduced to replace the label assignment of the native Task-Aligned Assigner in YOLOv8, achieving a one-to-one globally optimal assignment between predicted bounding boxes and ground-truth boxes, thus yielding more stable and accurate bounding box predictions in complex crack detection tasks. The loss function is mainly composed of three components: classification loss, bounding box regression loss and Generalized Intersection over Union (GIoU) loss. Based on the Hungarian Matching algorithm, the comprehensive cost between predicted bounding boxes and ground-truth boxes during the matching process is first calculated as follows:

2 $$Cost=\lambda _{{cls}}^{{match}} \cdot Cos{t_{cls}}+\lambda _{{bbox}}^{{match}} \cdot Cos{t_{bbox}}+\lambda _{{giou}}^{{match}} \cdot Cos{t_{iou}}$$

where $Cos{t_{cls}}$denotes the category prediction cost, $Cos{t_{bbox}}$the coordinate distance cost of bounding boxes, $Cos{t_{giou}}$the overlap cost of bounding boxes, and $\lambda _{{cls}}^{{match}}$, $\lambda _{{bbox}}^{{match}}$, $\lambda _{{giou}}^{{match}}$are the weight coefficients of each cost component, respectively.

The Hungarian Matching algorithm achieves a one-to-one optimal assignment between predicted bounding boxes and ground-truth boxes by solving the minimum weight matching of the cost matrix, ensuring that each ground-truth box is matched with the predicted bounding box with the lowest cost, and avoiding the redundancy problem of “multiple bounding boxes matching the same ground-truth box” in the native strategy. Based on the matching results, only the optimally matched predicted bounding boxes are used to calculate the loss, and the rest are treated as negative samples, which avoids gradient confusion caused by loss sharing.

For classification, a Focal Loss - based classification function is adopted to alleviate class imbalance, which can be expressed as:

3 $${L_{{\mathrm{cls}}}}=\frac{1}{N}\sum\limits_{{i=1}}^{N} {FL({p_i})}$$

where ${p_i}$ denotes the predicted category score, and FL represents the Focal Loss.

4 $$FL({p_i})= - \alpha {(1 - {p_i})^\gamma }\log ({p_i}) - (1 - \alpha ){p_i}^{\gamma }\log (1 - {p_i})$$

where $\alpha$ is the balance coefficient of the Focal Loss with a value of 0.25, $\gamma$ is the focal coefficient with a value of 2.

Bounding Box Regression Loss (fine-grained coordinate regression):

5 $${L_{{\mathrm{bbox}}}}=\frac{1}{N}\sum\limits_{{i=1}}^{N} {{{\left\| {{{\hat {b}}_i} - {b_i}} \right\|}_1}}$$

where ${\hat {b}_i}$ denotes the matched predicted bounding box, ${b_i}$ the ground-truth box, and ${\left\| {} \right\|_1}$ represents the L1 norm.

GIoU Loss (global position optimization):

6 $${L_{{\mathrm{GIoU}}}}=\frac{1}{N}\sum\limits_{{i=1}}^{N} {(1 - GIoU({{\hat {b}}_i} - {b_i}))}$$

where $GIoU({\hat {b}_i} - {b_i})$ denotes the Generalized Intersection over Union between the predicted bounding box ${\hat {b}_i}$ and the ground-truth box ${b_i}$.

The total loss is defined as:

7 $${L_{{\mathrm{total}}}}={\lambda _{{\mathrm{cls}}}}{L_{{\mathrm{cls}}}}+{\lambda _{{\mathrm{bbox}}}}{L_{{\mathrm{bbox}}}}+{\lambda _{{\mathrm{GIoU}}}}{L_{{\mathrm{GIoU}}}}$$

where ${\lambda _{{\mathrm{cls}}}}$ is the weight of the classification loss, ${\lambda _{{\mathrm{bbox}}}}$ the weight of the bounding box regression loss, and ${\lambda _{{\mathrm{GIoU}}}}$ the weight of the GIoU loss.

By incorporating the Hungarian Matching mechanism, the proposed method achieves more accurate and stable bounding box regression under set-based matching constraints, thereby enhancing both detection accuracy and model robustness.

### Evaluation metrics

To comprehensively assess the detection performance of the proposed model, several widely used evaluation metrics were employed, including Precision, Recall, F1-score, and mean Average Precision (mAP).

- Precision measures the accuracy of the model’s positive predictions.
- Recall evaluates the completeness of detected targets.
- F1-score represents the harmonic mean of Precision and Recall, balancing their trade-off.
- mAP is a comprehensive metric that calculates the average precision (AP) for each class at different Intersection over Union (IoU) thresholds and then averages over all classes.

The definitions of these metrics are as follows:

Precision:

8 $${\mathrm{Precision}}=\frac{{TP}}{{TP+FP}}$$

where TP denotes the number of true positives (correctly predicted positive samples), and FP denotes the number of false positives (incorrectly predicted positive samples).

Recall:

9 $${\mathrm{Recall}}=\frac{{TP}}{{TP+FN}}$$

where FN is the number of false negatives (missed positive samples).

F1-score:

10 $${\mathrm{F1}}=\frac{{2 \times {\mathrm{Precision}} \times {\mathrm{Recall}}}}{{{\mathrm{Precision}}+{\mathrm{Recall}}}}$$

Mean Average Precision (mAP):

11 $${\mathrm{mAP}}=\frac{1}{N}\sum\limits_{{i=1}}^{N} {{\mathrm{A}}{{\mathrm{P}}_i}}$$

where *N* is the total number of classes, and $A{P_i}$ is the average precision of the *i*-th class computed at different IoU thresholds. In this Research, both mAP@0.5 (IoU threshold = 0.5) and mAP@0.5:0.95 (averaged over IoU thresholds from 0.5 to 0.95 with a step size of 0.05) are reported to provide a comprehensive evaluation of the model’s detection performance.

## Training results analysis

**Fig. 10:**
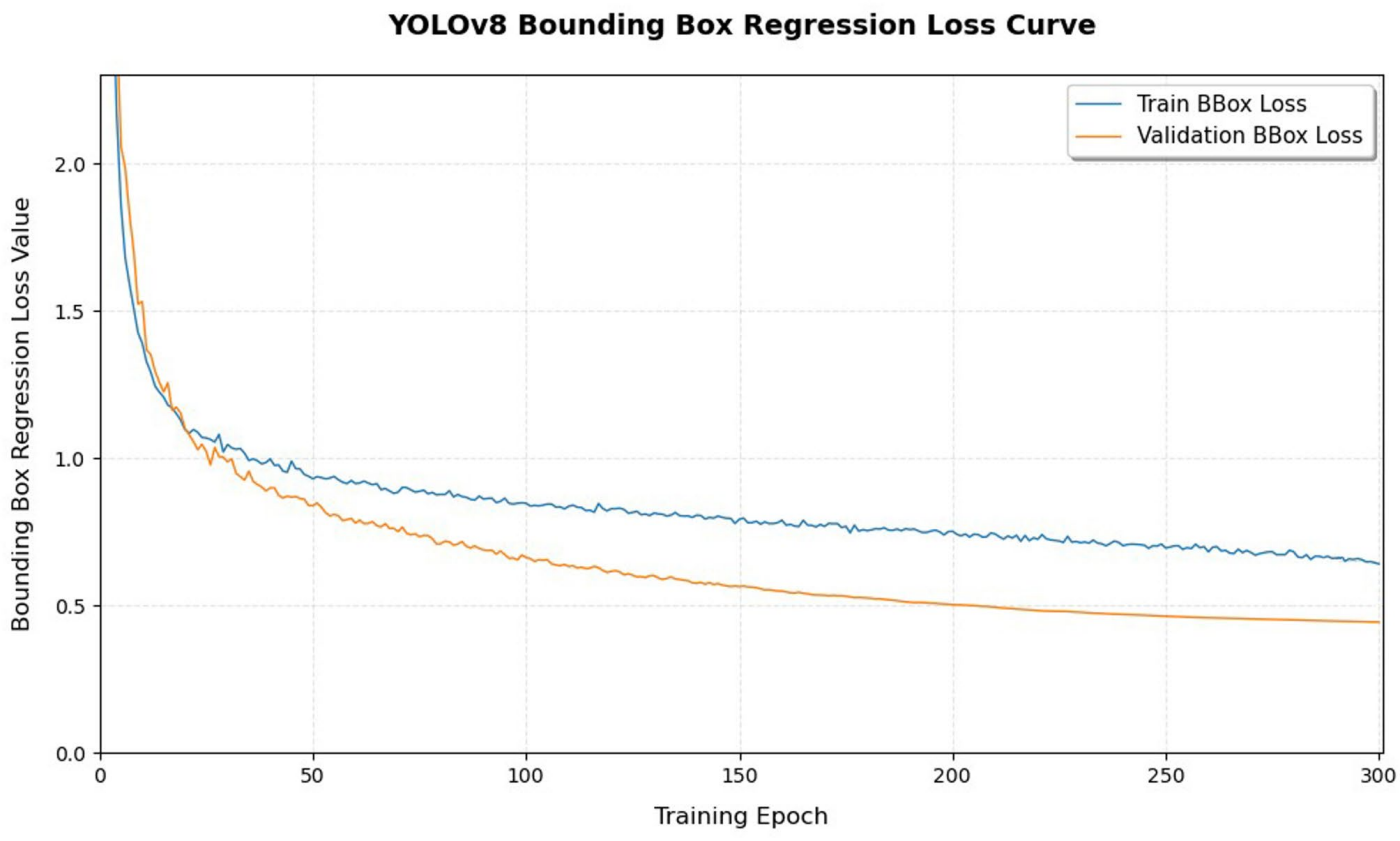
Bounding box regression loss curve.

During model training, the loss function trends and the improvement of key evaluation metrics were closely monitored. The training loss curve is shown in Fig. 10. Experimental results indicate that the total loss decreases rapidly during the early stages and gradually stabilizes after approximately 135 epochs, suggesting that the model has effectively learned features and achieved parameter convergence.

**Fig. 11:**
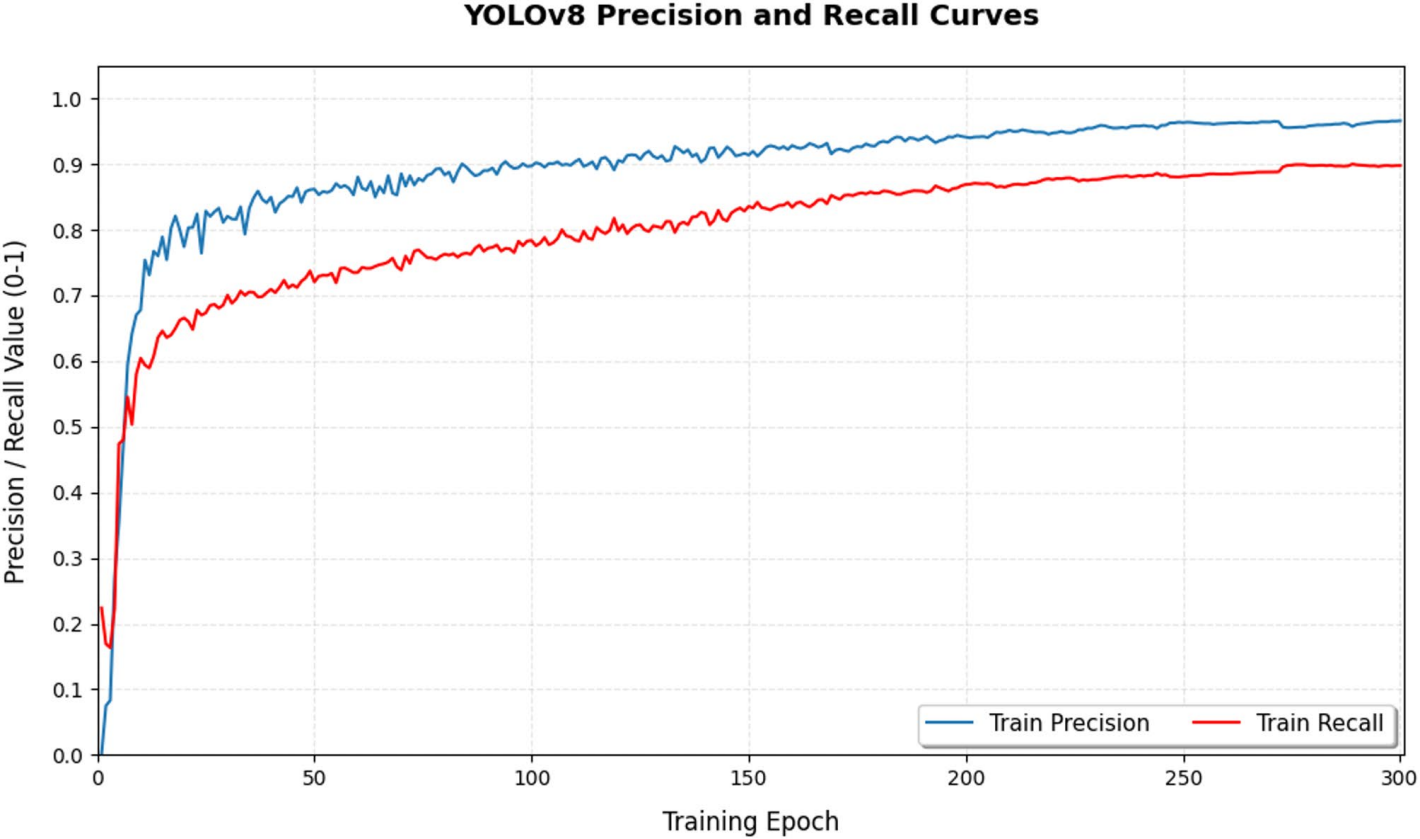
Precision–Recall variation curves.

Regarding the training and validation metrics, both Precision and Recall show a steady increase, eventually stabilizing at high levels, indicating that the model can balance accuracy and completeness in crack detection. Meanwhile, mAP@0.5 and mAP@0.5:0.95 continuously improve during training, eventually converging to optimal values, confirming that the proposed model performs well across different IoU thresholds. Compared with baseline models, EVA-YOLOv8 demonstrates faster convergence and higher stability during training (Fig. 11).

## Comparative experiments

### Comparison with existing methods

To verify the effectiveness of the proposed model, this research conducted comparative experiments with several mainstream object detection methods, including YOLOv5, YOLOv7, YOLOv9, YOLOv10, YOLOX-Nano, Faster R-CNN, and RT-DETR. All comparative models were trained from scratch on the same dataset and adopted entirely consistent training protocols as EVA-YOLOv8, covering data partitioning, data augmentation strategies, hyperparameter settings, training epochs and other aspects, so as to ensure the fairness and scientificity of the comparative experiments. The evaluation metrics included Precision, Recall, mAP@0.5, and mAP@0.5–0.95, while model complexity indicators (Params, FLOPs) were also considered for a comprehensive performance assessment.

As shown in Table 2, the two-stage detector Faster R-CNN exhibits relatively low detection accuracy with high parameter and computational overhead. RT-DETR, benefiting from the Transformer architecture, achieves strong global modeling capability but incurs a substantial computational cost. YOLOv7 and YOLOX-Nano offer faster inference speed but suffer from limited detection accuracy, whereas YOLOv5 maintains a relatively balanced trade-off between accuracy and efficiency. YOLOv9 and YOLO11 exhibit slightly lower detection performance compared with the proposed EVA-YOLOv8, while achieving a favorable compromise between computational cost and inference speed. YOLOv10, on the other hand, involves a relatively higher number of parameters and computational complexity. In contrast, the proposed EVA-YOLOv8 demonstrates superior detection performance among the compared models. achieving an mAP@0.5 of 0.897, mAP@0.5–0.95 of 0.706, Precision of 0.907, and Recall of 0.875, with only 3.59 M parameters and 7.5 GFLOPs. These results confirm the model’s advantages in lightweight design and engineering deployability.

**Fig. 12:**
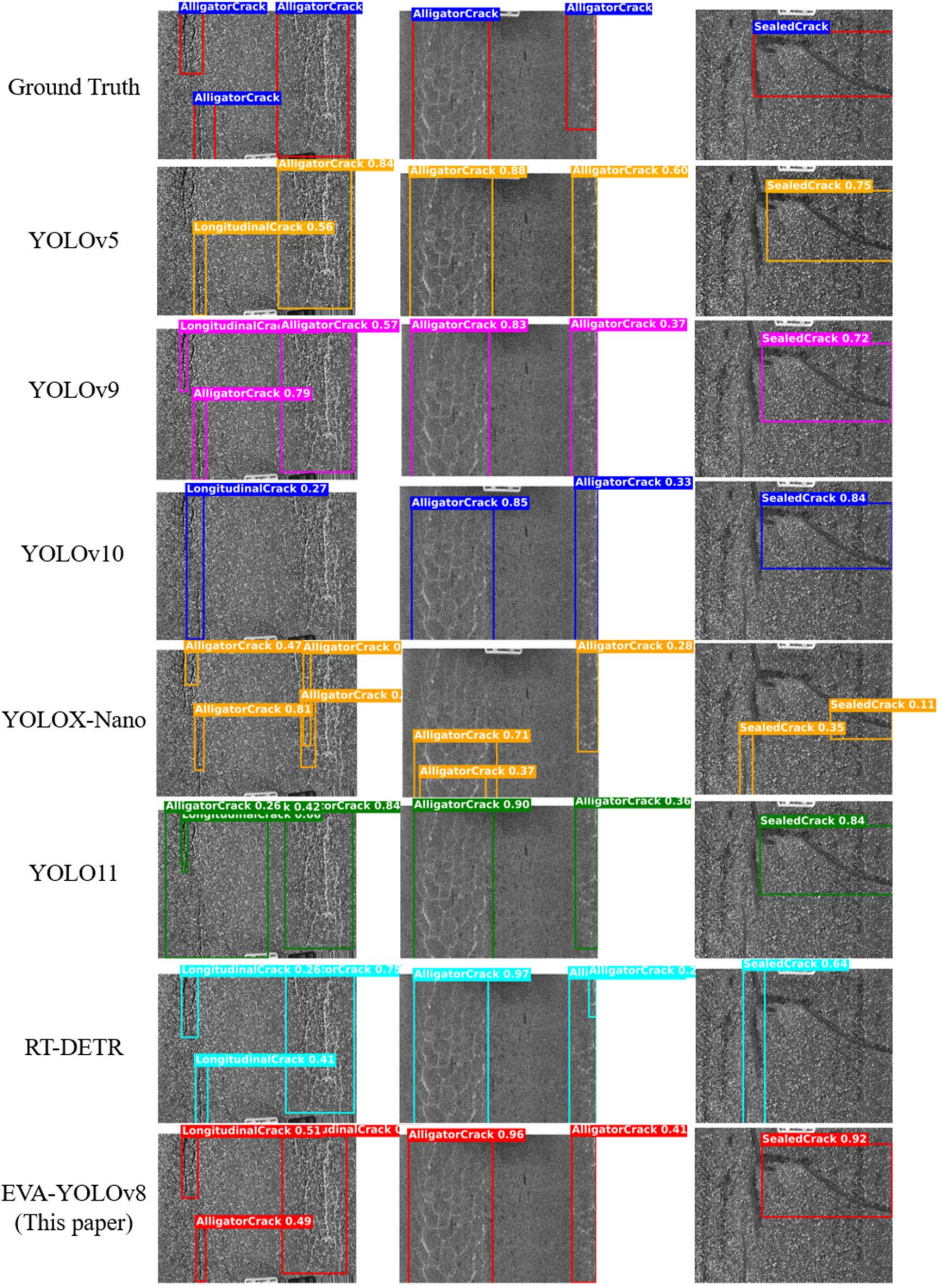
Comparison of detection results of different models.

As illustrated in Fig. 12, the detection results on representative crack samples further highlight the superiority of EVA-YOLOv8. Conventional models tend to miss or misidentify cracks in fine, shadowed, or low-contrast regions, whereas EVA-YOLOv8 more accurately focuses on crack areas, effectively suppresses back-ground interference, and maintains coherent detection boundaries, demonstrating stronger feature representation and robustness.

**Table 2:** Results of comparative experiments.

Model	mAP@0.5	mAP@0.5:0.95	Precision	Recall	F1	Params/M	GFLOPs	FPS
YOLOv5	0.877	0.656	0.897	0.851	0.873	7.021	15.8	239
YOLOv7	0.781	0.496	0.741	0.783	0.761	6.022	13.2	136
YOLOv9	0.87	0.653	0.844	0.831	0.837	9.785	39.8	92
YOLOv10	0.862	0.676	0.865	0.861	0.863	16.455	63.4	75
YOLOX-Nano	0.69	0.323	0.690	**0.919**	0.788	**0.9**	**2.55**	**392**
YOLO11	0.879	0.678	0.866	0.863	0.864	2.583	6.3	263
Faster R-CNN	0.863	0.658	0.817	0.816	0.816	5.243	20.7	95
RT-DETR	0.873	0.696	0.895	0.854	0.874	31.992	108.0	76
EVA-YOLOv8 (This paper)	**0.897**	**0.706**	**0.907**	0.881	**0.894**	3.006	7.5	208

Significant values are in bold.

### Cross-dataset evaluation

**Fig. 13:**
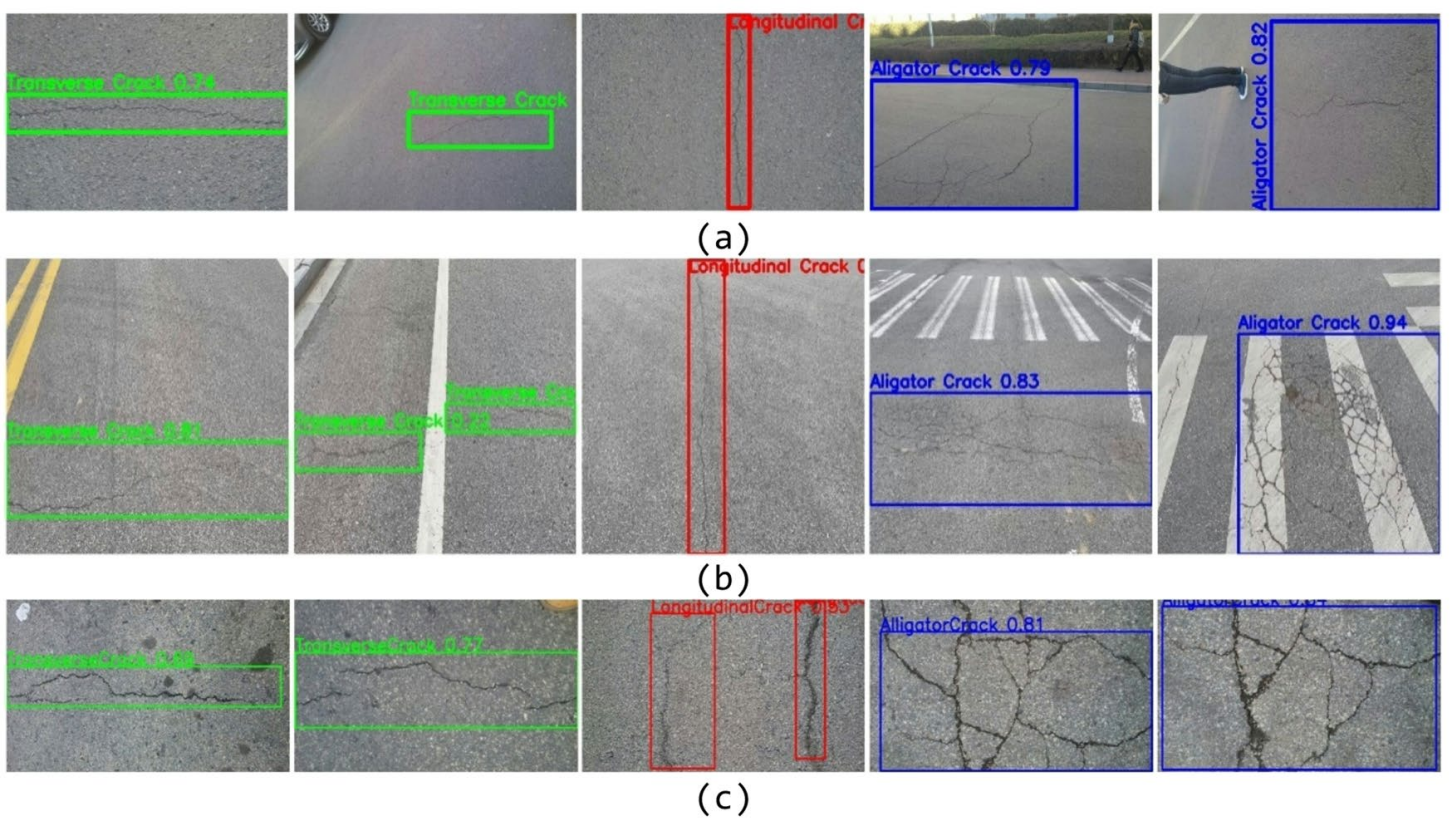
Visualization Results of EVA-YOLOv8 on Public Datasets. (**a**) Crack500 dataset, (**b**) RDD2022 dataset, (**c**) CFD dataset.

To evaluate the cross-dataset generalization capability of the proposed model, three publicly available crack detection datasets, Crack500^31^, RDD2022^32^, and CFD^33^, were selected for testing. The model was trained exclusively on the self-constructed dataset, and no samples from the public datasets were used for fine-tuning or parameter adjustment during the testing phase. Representative prediction results are illustrated in Fig. 13.

The three datasets exhibit notable differences in acquisition environments, imaging conditions, and crack morphologies, which pose challenges to the model’s generalization ability. In the experiments, the trained model was directly applied to each public dataset for inference, and the detection results were analyzed through qualitative visualization.

The results indicate that the proposed model can accurately localize crack regions across different datasets and maintains stable detection performance under significant variations in scale and background complexity. However, in a limited number of cases, such as in the RDD2022 dataset, cracks with extremely small scales or high visual similarity to background textures may not be fully detected. This suggests that the representation of fine-grained features for small-scale cracks still has room for improvement in cross-dataset evaluation.

## Ablation experiments

To further validate the effectiveness of key modules in the proposed method, a se-ries of ablation experiments were conducted. The experiments focused on the intro-duction of the MobileViT Block module, the Efficient Multi-scale Attention (EMA) mechanism, and the GhostConv convolution module.

### Analysis of ablation experiment results

Different modules were gradually integrated into the baseline YOLOv8 model. The performance of all resulting models, evaluated under identical experimental con-ditions, is presented in Table 3.

**Table 3:** Evaluation metrics of models in ablation experiments.

Model	MobileViT Block	EMA	Ghost Conv	mAP@0.5	mAP@ 0.5:0.95	Precision	Recall	F1	Params/M	GFLOPs	FPS	AUC
Baseline (YOLOv8n)				0.831	0.623	0.862	0.828	0.845	3.59	8.1	232	0.782
YOLOv8-V	✓			0.872	0.649	0.876	0.853	0.864	3.796	8.5	212	0.767
YOLOv8-E		✓		0.876	0.655	0.856	0.865	0.860	3.349	8.7	210	0.839
YOLOv8-G			✓	0.877	0.668	0.879	0.844	0.861	**2.726**	**6.4**	**245**	0.723
YOLOv8-VE	✓	✓		0.894	0.685	0.876	0.875	0.875	4.139	9.2	186	0.867
EVA-YOLOv8 (This paper)	✓	✓	✓	**0.897**	**0.706**	**0.907**	**0.881**	**0.894**	3.006	7.5	208	**0.895**

Significant values are in bold.

Specifically, based on the original YOLOv8, different modules were progressively added and evaluated under identical experimental conditions.

1. Impact of MobileViT Block:
  Incorporating the MobileViT Block into the baseline model increased mAP@0.5 by approximately 4.1% and mAP@0.5–0.95 by about 2.6%, indicating that the global modeling capability of MobileViT enhances feature representation, especially under complex back-grounds and for small targets. However, this also slightly increases the number of parameters and computational cost.
2. Impact of EMA mechanism:
  Adding the EMA module resulted in an mAP@0.5 increase of 4.5%, demonstrating that EMA effectively enhances multi-scale feature interaction and improves the model’s adaptability to variations in target size, thereby reducing false negatives and false positives.
3. Impact of GhostConv module:
  After introducing the GhostConv module, the number of model parameters was reduced by 864,000 and the computational cost decreased by 1.7 GFLOPs, resulting in an inference speed improvement of 13 FPS. Compared with the original YOLOv8 model, EVA-YOLOv8 achieves a 16.3% reduction in parameter count and a decrease of 0.6 GFLOPs, while the inference speed is reduced by 24 FPS. Meanwhile, the mAP performance remains nearly unchanged and even shows a slight improvement. These results indicate that the GhostConv module contributes to model lightweight design and computational efficiency without degrading detection accuracy.

In the combined experiments, MobileViT and EMA exhibit a clear synergistic effect. Compared with the configuration using only MobileViT, the combination of MobileViT and EMA improves mAP@0.5 by 2.2% and mAP@0.5:0.95 by 3.6%. Relative to the configuration using only EMA, mAP@0.5 and mAP@0.5:0.95 are increased by 1.8% and 3.0%, respectively. These results suggest that the integration of global modeling and multi-scale attention provides complementary information, leading to improved detection performance.

Overall, the introduction of GhostConv enables a more favorable trade-off between detection accuracy and computational efficiency, making the proposed model more suitable for mobile and edge deployment. The final configuration achieves mAP@0.5 and mAP@0.5:0.95 values of 89.7% and 70.6%, respectively.

### Analysis of Grad-CAM + + Heatmaps

Deep convolutional neural networks (CNNs) for object detection are often regarded as “black-box” models, as their internal decision-making processes are difficult to interpret. To enhance the interpretability of the YOLOv8 model, this research employs Grad-CAM++^34,35^ as the primary visualization tool. Grad-CAM + + is an improved version of Grad-CAM that incorporates higher-order gradient information to optimize the computation of feature importance weights, enabling more accurate and robust visual explanations in multi-object, overlapping, and fine-grained scenarios. The core idea of Grad-CAM + + is to determine the importance weights of convolutional feature maps by leveraging the gradients of the class prediction score ${y^c}$ with respect to the convolutional feature map ${A^k}$. The contribution weight $\alpha _{k}^{c}$ of feature map ${A^k}$ to class *c* is defined as:

12 $$\alpha _{k}^{c}=\frac{1}{{u \times v}}\sum\nolimits_{{i=1}}^{u} {\sum\nolimits_{{j=1}}^{v} {\frac{{\partial {y^c}}}{{\partial A_{{ij}}^{k}}}} }$$

Here, $\alpha _{k}^{c}$ denotes the contribution weight of the feature map to class *c*, while $\frac{{\partial {y^c}}}{{\partial A_{{ij}}^{k}}}$ represents the gradient of the class score with respect to the pixel at position (*i*, *j*) in the feature map.

where $u \times v$ denotes the spatial resolution of the feature map, ${A_{ij}}^{k}$ represents the activation value of the *k*-th feature map at spatial location (*i*, *j*), and $\frac{{\partial {y^c}}}{{\partial A_{{ij}}^{k}}}$ denotes the gradient of the class score with respect to the corresponding pixel location.

Subsequently, the weighted sum of the n feature maps is computed, and the Grad-CAM + + representation for class c is obtained by applying the ReLU operation as:

13 $$L_{{Grad - CAM++}}^{c}={\mathrm{ReLU}}(\sum\limits_{{k=1}}^{n} {\alpha _{k}^{c}{A_{ij}}^{k}} )$$

where the ReLU operation is applied to retain regions that contribute positively to the prediction. The resulting class activation heatmap provides an intuitive visualization of the key regions attended by the model during the prediction process, thereby offering interpretability support for the detection results.

To quantitatively evaluate the model’s attention to crack regions, the Area Under the ROC Curve (AUC) is used to quantify the matching degree between the heatmap and the ground-truth annotated bounding box. First, the Grad-CAM + + heatmap is normalized to the range [0,1], and a pixel-level binary mask is generated based on the ground-truth annotated bounding box, with pixels inside the box labeled as positive samples and those outside as negative samples. Then, the heatmap pixel values are taken as judgment scores to plot the ROC curve with FPR as the horizontal axis and TPR as the vertical axis, and the AUC value is finally calculated as follows:

14 $$AUC = \frac{1}{2}\sum\limits_{{n = 1}}^{{k - 1}} {\left( {FPR_{{n + 1}} - FPR_{n} } \right) \cdot \left( {TPR_{{n + 1}} - TPR_{n} } \right)}$$

15 $$FPR=\frac{{FP}}{{FP+TN}}$$

16 $$TPR=\frac{{TP}}{{TP+FN}}$$

where TP denotes the number of true positives, FP denotes the number of false positives, TN denotes the number of true negatives, and FN denotes the number of false negatives.

**Fig. 14:**
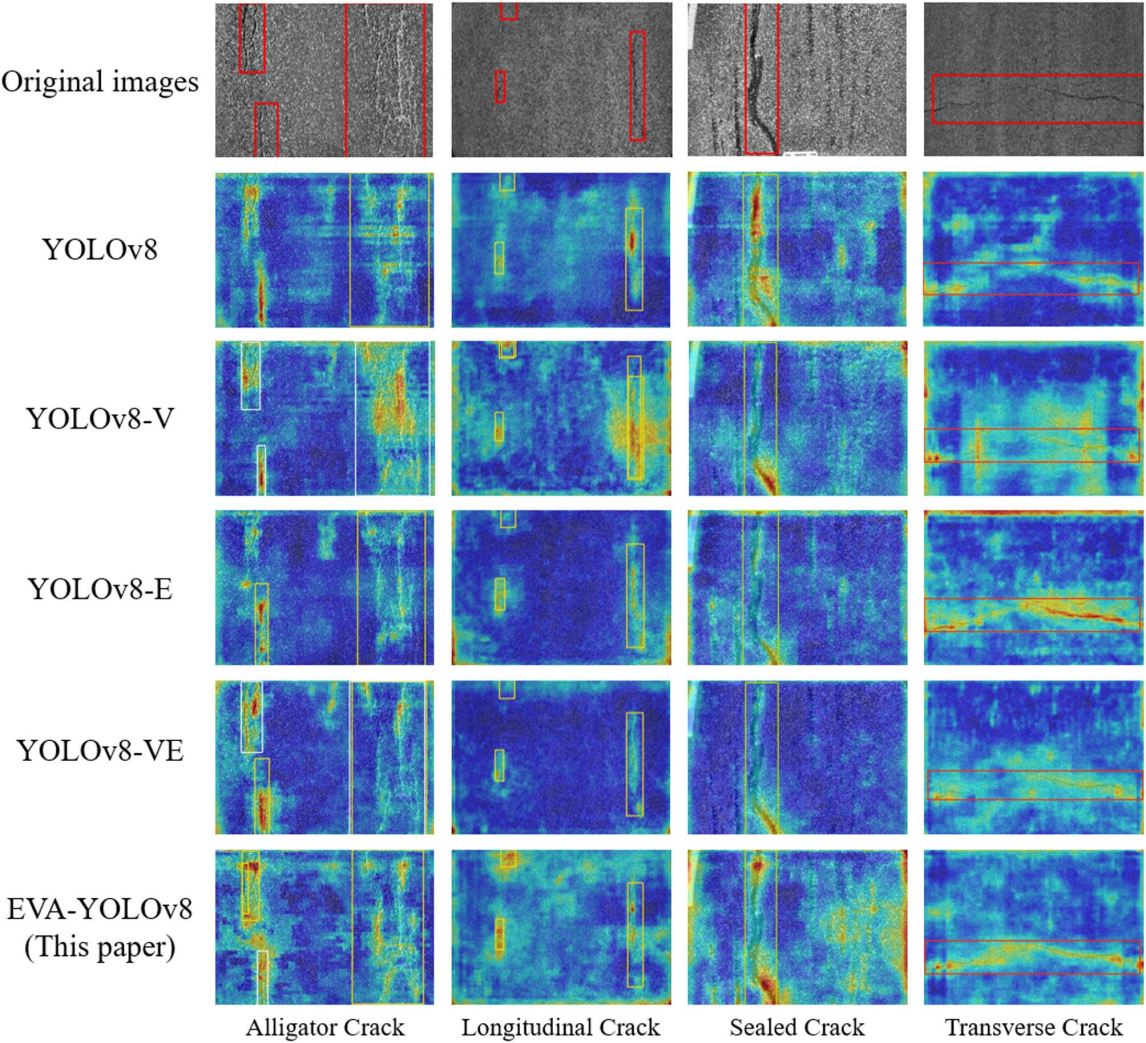
Grad-CAM + + heatmaps of different crack categories (Red regions correspond to the areas of highest model attention and are regarded as the most discriminative features during prediction, whereas yellow and green indicate moderate attention, and blue denotes regions considered irrelevant to the target.)

Based on the data in Table 3, it can be observed from Fig. 14 that the baseline model primarily responds to the most salient regions of the targets, exhibiting local feature overfitting and relatively weak responses to edges or small targets. After incorporating the MobileViT Block, the model captures a broader global contextual representation, with heatmaps showing a more uniform response across the entire target area, demonstrating enhanced global perception.

With the further integration of the EMA efficient multi-scale attention mechanism, the model exhibits more precise response distributions across targets of different scales and demonstrates strong robustness in complex environments.

Finally, the EVA-YOLOv8 model shows relatively clear target response features in the heatmaps. The model not only exhibits higher response values in the main target regions, but also performs well in suppressing the background and delineating target boundaries. This indicates that the combined structure improves the model’s perception and localization of targets in complex scenes while maintaining a lightweight design.

## Discussion

Although the proposed EVA-YOLOv8 model demonstrates superior performance in terms of detection accuracy, robustness, and computational efficiency, several limitations remain to be addressed in future work.

### Dataset diversity and scale limitations

The dataset covers representative pavement crack types but is limited in scale, with most samples collected under controlled conditions. Future work will include in situ samples from diverse climates and pavement types, as well as GAN-generated synthetic data, and will perform cross-dataset evaluation using public benchmarks such as UAV-PDD2023 to assess generalization.

### Dependence on RGB imagery

Reliance on RGB imagery makes the model sensitive to illumination changes and occlusion. Future studies will explore multimodal fusion with LiDAR (for depth) and infrared thermal imaging (for subsurface cracks) to enhance robustness.

### Lightweight deployment and computational trade-offs

Although GhostConv and efficient attention reduce parameters and FLOPs, real-time inference on low-power edge devices remains challenging. Further optimization via TensorRT quantization, structured pruning, or distillation will be explored to balance accuracy and deployability.

### Model interpretability and decision transparency

Grad-CAM + + provides qualitative attention visualizations but lacks quantitative evaluation of feature attribution and cross-layer consistency. Future work will investigate metrics such as Feature Attribution Scores (FAS) to improve interpretability and reliability.

## Conclusion

To address the limitations of traditional crack detection methods, such as low efficiency and limited generalization, this research proposes an improved YOLOv8-based model, EVA-YOLOv8(Efficient ViT Attention-YOLOv8), for multi-class and multi-scale pavement crack detection. The main conclusions are as follows:

1. Based on the YOLOv8n framework, EVA-YOLOv8 incorporates a MobileViT Block and an Efficient Multi-scale Attention (EMA) mechanism, enhancing the model’s feature representation capabilities. The proposed model achieved average values of 0.897 (mAP@0.5), 0.706 (mAP@0.5:0.95), 0.907 (Precision), 0.881 (Recall), and 0.894 (F1-score). These results demonstrate that the proposed approach better captures the global spatial dependencies of cracks, thereby improving the detection of fine and low-contrast cracks.
2. The experimental results show that EVA achieves strong performance across the main evaluation metrics while maintaining a lightweight design, with a 16.3% reduction in parameters and a 7.4% reduction in GFLOPs compared to the baseline model. Ablation studies confirm the key role of the MobileViT Block and EMA modules in improving detection accuracy. Comparative experiments further demonstrate that EVA-YOLOv8 achieves favorable performance in both accuracy and inference speed.
3. As revealed by the Grad-CAM + + heatmaps, the proposed EVA-YOLOv8 model exhibits activation in primary target regions and yields rational responses in object boundary perception and background suppression. This indicates that the designed hybrid structure enhances the model’s perceptual and localization capabilities in complex scenarios.

## Supplementary Information

Below is the link to the electronic supplementary material.

Supplementary Material 1

## Data Availability

Data are available from the corresponding author upon reasonable request.
