# Supplementary material for: EVA-YOLOv8: an improved YOLOv8 model integrating multi-scale attention mechanism and vision transformer for multi-class road crack detection
Source: Sci Rep. 2026 Apr 6;16:16504. doi: 10.1038/s41598-026-46475-0 (PMC13216579; doi:10.1038/s41598-026-46475-0)
Supplement: Supplementary file 1 — Supplementary Material 1 [file 41598_2026_46475_MOESM1_ESM.pdf]

To ensure a fair comparison and reproducibility, all models were trained and evaluated under identical experimental settings.

Training hyperparameters and implementation details for all compared models

**Category**	**Parameter**	**Value**
Framework & Hardware	Framework	PyTorch 2.5.1
	GPU	NVIDIA RTX 4070 Super
	CUDA	11.8
Input Settings	Input image size	960×960
	Batch size	32
	Epochs	300
Optimization	Initial learning rate	0.01
	Weight decay	0.0005
	Momentum	0.937
Data & Augmentation	Dataset split	Train/Val/Test 3441/1070/541
	Data augmentation	Photometric, geometric, random erasing, mosaic
